# Health-related knowledge and preferences in low socio-economic kindergarteners

**DOI:** 10.1186/1479-5868-9-1

**Published:** 2012-01-10

**Authors:** Dan Nemet, Deganit Geva, Yoav Meckel, Alon Eliakim

**Affiliations:** 1 Child Health and Sports Center, Pediatric Department, Meir Medical Center, Kfar-Saba, Sackler School of Medicine, Tel-Aviv University, Israel; 2 Zinman College of Physical Education, Wingate Institute, Netanya, Israel

**Keywords:** knowledge, low socio-economic, nutrition, questionnaires, physical activity, preferences, pre-school

## Abstract

**Objective:**

The aim of the present study was to determine physical activity (PA) and nutrition knowledge and preferences in low socio-economic status kindergarten children.

**Methods:**

Following height and weight measurement, 795 low socio-economic status kindergarten children (age 3.8-6.8 y.o) completed a photo-pair knowledge and preferences food and exercise questionnaire.

**Results:**

No difference was found between nutrition and PA knowledge scores (52.3 ± 0.9 versus 52.6 ± 0.8%, respectively). There was no difference between the nutrition knowledge and preference score (52.3 ± 0.9 versus 50.9 ± 0.9%, respectively). PA preference was significantly higher than knowledge (56.9 ± 1.5 versus 52.6 ± 0.8%, respectively; p < 0.0001). Significant correlations were found between nutrition knowledge and preferences (r = 0.55, p < 0.0001), physical activity knowledge and preferences (r = 0.46, p < 0.0001), and nutrition and PA preferences (r = 0.46, p < 0.001). Nutrition preference scores were significantly lower in overweight compared to normal weight kindergartners 48.1 ± 1.7 versus 52.0 ± 1.0%; p < 0.05). PA knowledge and preference scores were significantly higher among male compared to the female kindergartners (p < 0.001 for both).

**Conclusion:**

Our data demonstrate diversities in physical activity and nutrition knowledge and preferences among low socio-economic status kindergarten children. These findings may be important for the development of health promotion programs in low socioeconomic kindergarten children.

## Introduction

Despite major efforts to prevent weight gain or to promote weight reduction, the prevalence of childhood obesity increases in epidemic proportions throughout Westernized societies [1]. Children who are obese in their preschool years are more likely to become obese adolescents and adults [2] and to develop diabetes, hypertension, hyperlipidemia, asthma, and sleep apnea. One of the USA Healthy People 2010 objectives was therefore to reduce to 5% the proportion of children and adolescents who are obese (http://www.HealthyPeople.gov). In the USA, obesity prevalence among low-income, preschool-aged children increased steadily from 12.4% in 1998 to 14.5% in 2003, and remained essentially the same, with a 14.6% prevalence in 2008 [3]. However, studies in the New-York city public elementary schools (mainly of low income families) reported a much higher overall overweight (BMI 85th to < 95th percentile) prevalence rate of 48%, with a 23% prevalence rate of obesity (BMI ≥ 95%ile) in kindergartners [4]; and studies have shown a significantly higher prevalence among Hispanic compared to African-Americans and Caucasian children [4,5]. This indicates that preventive and therapeutic interventions should start from at least as early as the pre-school years.

The causes for the increasing prevalence of childhood obesity are not completely understood, but life-style changes associated with increased caloric intake and decreased energy expenditure may play an important role, especially in genetically predisposed populations [1,6,7]. This indicates that a multi-disciplinary approach, that include life-style/behavioral modification, nutrition education and changes in physical activity patterns [8,9] should be used for preventive health education and therapeutic programs of childhood obesity. The design of such programs must take into account the existing nutrition and physical activity knowledge and preferences of children, particularly for those in pre-school age.

To date, very few studies examined the nutrition knowledge of elementary school-aged and kindergarten children. Studies in school-children raised concerns that lack of knowledge about food composition, inability to choose foods low in fat and/or saturated fat, and limited understanding of fiber [10,11]. We previously demonstrated in kindergarten children from moderate-high socio-economic classes that while nutrition knowledge was significantly higher than physical activity knowledge, nutrition preferences were not consistent with the knowledge, but physical activity knowledge and preference were found to be consistent. Moreover, gender-specific differences indicated better nutrition knowledge in girls and better physical activity preferences in boys [12]. Some studies [13] but not all [14], suggest that in school aged children obesity and health habits are closely related to knowledge. Yet, the relationship between nutrition and physical activity knowledge and preferences and the development of childhood obesity in kindergarten children has not been established.

Childhood obesity prevalence is increasing in low socio-economic class compared to the general population, and disparities in pediatric overweight along ethnic and socio-economic lines are expected to further exacerbate current disparities in rates of chronic diseases [15]. Therefore, there is a great need to study the nutrition and physical activity knowledge and preferences in this population. Thus, the aim of the present study was to determine the nutrition and physical activity knowledge and preferences in low socio-economic Israeli kindergarten children, and to compare them to our previous results from moderate-high socio-economic kindergarten children. We hypothesize that: 1. The prevalence of childhood obesity would be higher in low compared to moderate-high socio-economic kindergartners; 2. The and physical activity knowledge scores would be lower in low compared to moderate-high socio-economic kindergartners; 3. Nutrition preferences scores of the low socio-economic children would differ from their knowledge scores; 4. Nutrition and physical activity knowledge and preferences would be lower in overweight compared to normal weight kindergartners; and 5. Nutrition knowledge and preferences would be higher in females and physical activity preferences would be higher in males.

## Subjects and Methods

Seven hundred and ninety-five healthy kindergarten children (mean age 5.20 ± 0.02 years; 53% males; 47% females) participated in the study. Two hundred and twenty-three children (28.0%) had body mass index (BMI) equal to or above the 85^th ^percentile and were defined as overweight and obese, of them, 79 children (9.9%) had body mass index (BMI) equal to or above the 95^th ^percentile and were defined as obese, and 144 (18.1%) had BMI of equal to or above 85 to < 95th percentile, and were defined as overweight [16]. The participants came from 30 different kindergarten classes, from three low socio-economic status neighborhoods in the Sharon area in Israel. The study was approved by the Institutional Review Board of the Meir Medical Center, and all of the participants' parents signed an informed consent.

### Anthropometric measurements

Standard, calibrated scales and stadiometers were used to determine height, and weight. Child BMI (kg/m^2^) was calculated, and since BMI differs by age and gender, BMI-for-age and gender percentile was calculated according to the Center for Disease Control growth charts [17].

### Nutrition and physical activity knowledge and preferences evaluation

Nutrition and physical activity knowledge and preferences were evaluated by a photo-pair food and exercise questionnaire based on a questionnaire developed by Calfas et al. [18]. At the beginning of the test children were asked if they knew what healthy means, and they were provided with an age appropriate explanation, such as "Being healthy means that you can play outside, you do not get sick, and you feel good." To determine knowledge, children were asked to choose a doll which they were supposed to help stay healthy and take care of. The doll was used so that the child would assume a more caretaking approach and to make it less likely that they make choices purely based on personal preferences. Then children were presented with 15 photo-pairs; in each set of pictures, presented on a laptop computer, one picture signified the healthy choice and the other the unhealthy one. Children were asked to identify which food/activity of the pair would "make the doll healthy and grow big and strong."

To determine preferences, children were presented with the same 15 pairs of pictures and were asked to point to the food/activity they best like. This type of visual instrument was previously found to be appropriate for this age group and was validated on similar groups of children in the United States [18]. The order of knowledge or preferences testing was randomly assigned using a computerized program. A score (0-100%), representing the percentage of 'healthy' option's chosen by the children out of 15 photo-pairs was calculated and used for statistical analysis.

We compared the nutrition and physical activity knowledge and preference scores of the participants in the present study with our previously published data of kindergarten children (n = 202) from moderate-high socio-economic status living in the same geographic region (Sharon area) [12].

### Statistical analysis

Paired t-test was used to determine differences between nutrition and physical activity knowledge and preferences. Two sample t-test was used to determine differences in nutrition and physical activity knowledge and preferences between male and female subjects, and between overweight and normal weight children. Two sample t-test was also used to determine differences in nutrition and physical activity knowledge and preferences between kindergarten children from low or moderate-high socio-economic status. Pearson correlations were used to evaluate the relationship between nutrition knowledge and preferences and physical activity knowledge and preferences. Statistical significance was determined at the p < 0.05 level. Data are presented as mean ± standard error of the mean (SEM).

## Results

**Figure 1:**
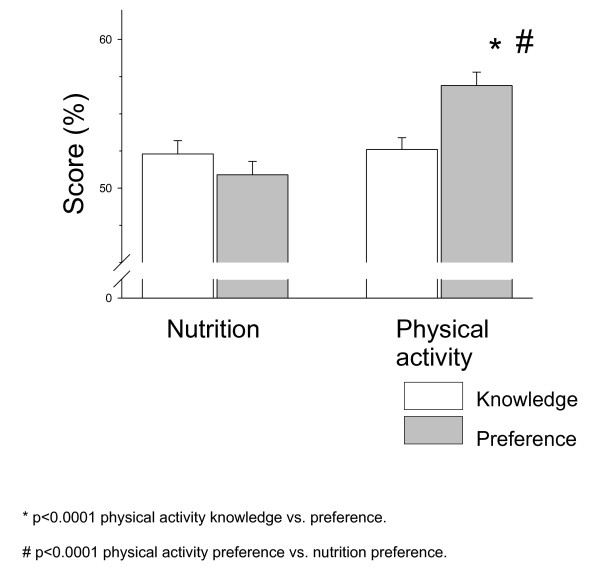
**Nutrition and physical activity knowledge and preferences of low socio-economic status kindergarten children**.

Anthropometric characteristics of the study participants are summarized in Table 1. There was no difference between the kindergarten children's nutrition and physical activity knowledge scores (52.3 ± 0.9 versus 52.6 ± 0.8%, respectively). There was no difference between the nutrition knowledge and preference score (52.3 ± 0.9 versus 50.9 ± 0.9%, respectively). Physical activity preference was significantly higher than physical activity knowledge (56.9 ± 1.5 versus 52.6 ± 0.8%, respectively; p < 0.0001), and significantly higher than nutrition preferences (56.9 ± 1.5 versus 50.9 ± 0.9%, respectively; p < 0.0001; Figure 1) among the kindergarten children.

**Table 1:** Descriptives of 795 low socioeconomic kindergarten children.

Age (years)		5.20 ± 0.02
Gender (M/F)	%	53/47

	n	422/373

Weight (kg)		19.3 ± 0.1

Height (cm)		108.9 ± 0.2

BMI (kg/m^2^)		16.20 ± 0.10

BMI Percentile		62.4 ± 1.0

Normal weight		72% n = 572

Obese	BMI%ile ≥ 95	9.9% n = 79

	BMI%ile ≥ 85	28.0% n = 223

Data are reported as Mean ± SEM

BMI = Body Mass Index

**Figure 2:**
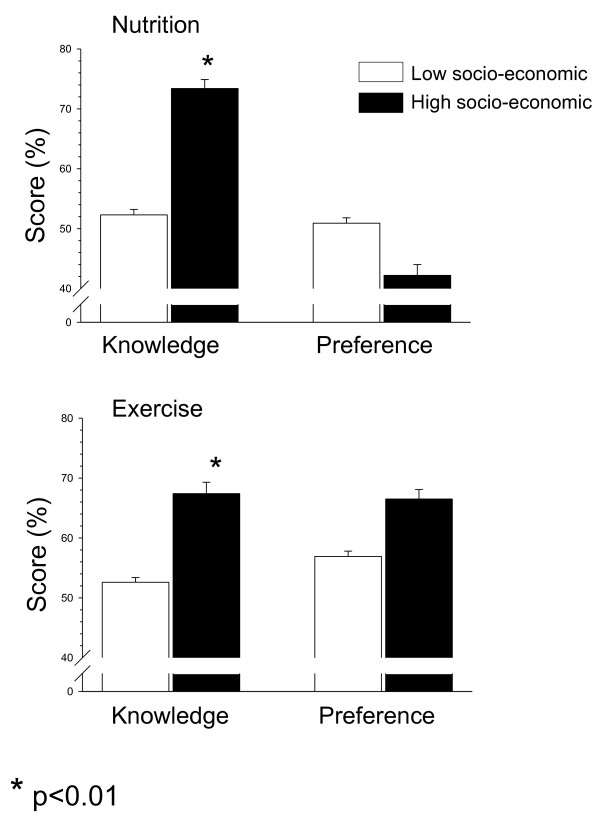
**Differences in nutrition and physical activity knowledge and preferences between low and moderate-high socio-economic kindergarten children**.

Nutrition and physical activity knowledge of the kindergarten children from low socio-economic status in the present study were significantly lower compared to those of moderate-high socio-economic status 202 kindergarten children from the same geographic region (Sharon area), (nutrition: 52.3 ± 0.9 versus 73.4 ± 1.5%, p < 0.01; exercise: 52.6 ± 0.8 versus 67.4 ± 1.9%, p < 0.01). There was no significant difference between the nutrition and physical activity preferences between kindergarten children from low and moderate-high socio-economic status (Figure 2).

There was a significant correlation between the scores of nutrition knowledge and preferences (r = 0.55, p < 0.0001), and between the scores of physical activity knowledge and preferences (r = 0.46, p < 0.0001). There was also a significant correlation between nutrition and physical activity preference scores (r = 0.46, p < 0.0001), and between nutrition and physical activity knowledge scores (r = 0.25, p < 0.001).

**Figure 3:**
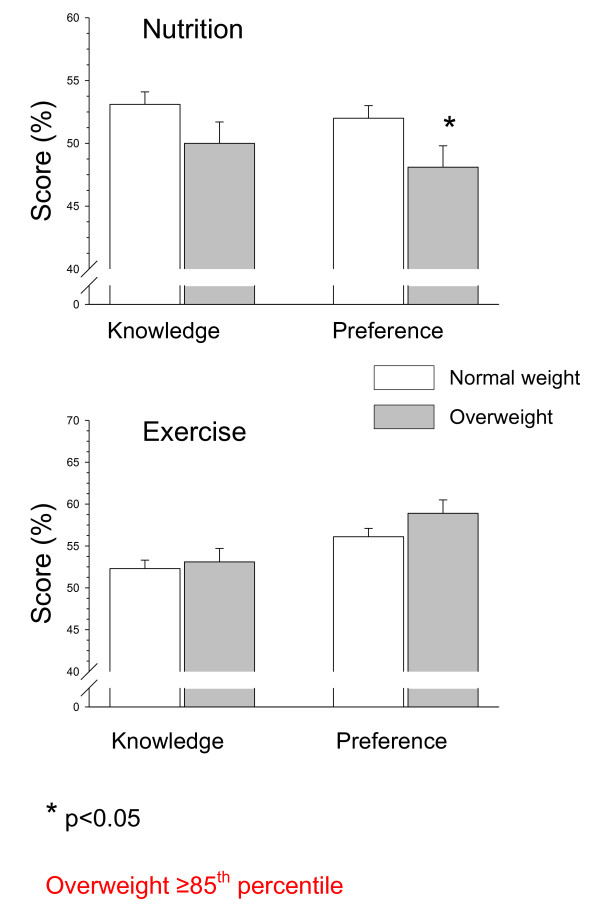
**Differences in nutrition and physical activity knowledge and preferencesbetween normal weight and overweight low socioeconomic kindergarten children**.

Nutrition preferences were significantly lower among the overweight compared to the normal weight kindergartners (48.1 ± 1.7 versus 52.0 ± 1.0%, respectively; p < 0.05). No significant differences in nutrition knowledge, and physical activity knowledge or preferences scores were found between the overweight and normal weight kindergartners (Figure 3).

**Figure 4:**
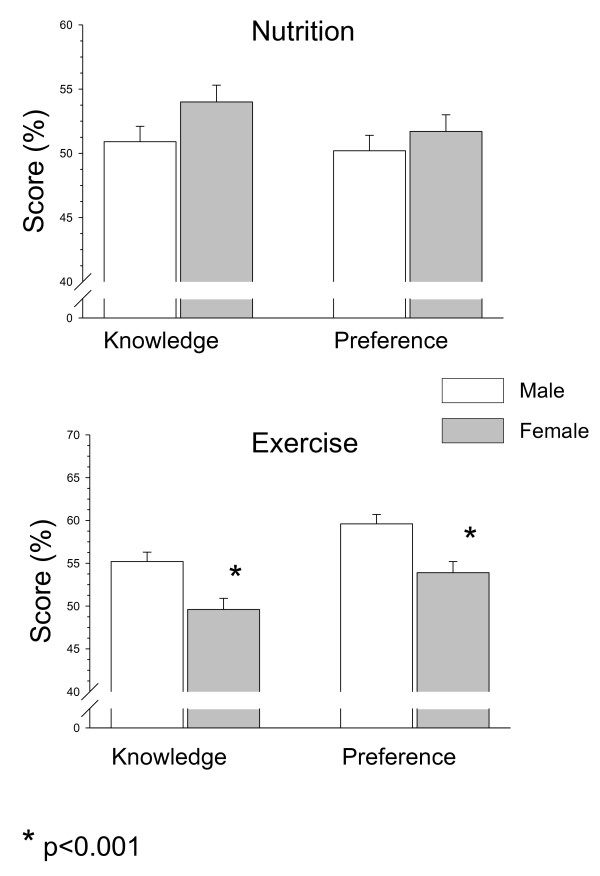
**Differences in nutrition and physical activity knowledge and preferences between female and male low socioeconomic kindergarten children**.

There were no significant gender differences in nutrition knowledge and preferences, although female kindergartners showed a tendency for higher nutrition knowledge scores compared to the male kindergartners (p < 0.08; Figure 4). Physical activity knowledge and preference scores were significantly higher among male compared to the female kindergartners (p < 0.001 for both, Figure 4).

In both genders physical activity preference was higher than physical activity knowledge (males: 59.6 ± 1.1 versus 55.2 ± 1.1, p < 0.0001; females: 53.9 ± 1.3 versus 49.6 ± 1.3, p < 0.001; Figure 4). In the females, nutrition knowledge was higher than physical activity knowledge (54.0 ± 1.3 versus 49.6 ± 1.3, p < 0.01), while in the males, physical activity knowledge was higher than the nutrition knowledge (55.2 ± 1.1 versus 50.9 ± 1.2, p < 0.005; Figure 4).

## Discussion

Childhood obesity has reached epidemic proportions in recent years, and the prevalence of childhood obesity in Israel is among the highest compared to European countries and the United States of America [19]. In our present cohort of kindergarten children from low socio-economic status in Israel, the overall prevalence of overweight (BMI ≥ 85%ile) was 28%, with 10% of children found to be obese (BMI ≥ 95%). This rate of overweight and obesity is a significantly higher than the expected value of 15% (above the 85^th ^percentile), and higher from our previous report of 22% in moderate-high socio-economic status kindergarten children from the same area [12]. This suggests that childhood obesity is an important public health concern from very early stages of life, and that preventive and therapeutic interventions must start, therefore, as early as the pre-school years especially in low socio-economic classes.

The mechanism for the increased prevalence of childhood obesity is not clear, but probably includes continued imbalance between increased caloric intake and reduced energy expenditure. Therefore, understanding the nutrition and physical activity knowledge and preferences of children is crucial for the development of strategies for the prevention and treatment of childhood obesity.

The present study raises several interesting issues. First, we found that the nutrition and physical activity knowledge is poor among low socio-economic kindergarten children (~52%), and significantly reduced compared to moderate-high socio-economic kindergarten children (Figure 2). This may explain, at least partially, the higher prevalence of overweight and obesity in the low socio-economic status kindergarten children. These results emphasize the importance of parents, home and the natural environment for the general knowledge during childhood, since topics of nutrition and physical activity and their health-related benefits are not taught in kindergarten and preschools. Interestingly, the physical activity preferences of the low SES children were significantly higher than their physical activity knowledge. While this may partially compensate for the lack of knowledge, physical activity preferences were still lower compared to that of moderate-high kindergarten children (Figure 2).

We previously demonstrated that despite the good nutrition knowledge in moderate-high socio-economic kindergarten children, their nutrition preferences scores were significantly lower. This was in agreement with a previous study in kindergarten children which also reported inconsistency between food preferences and knowledge of the dietary guidelines [11]. These results suggest that dietary education should not only focus on nutrition information, but should introduce new ways to implement the existing knowledge in order to make better and healthier food choices and preferences. However, for the low socio-economic status kindergarten children poor nutrition knowledge scores, along with the significant correlation between nutrition knowledge and preferences suggest that the main focus of nutrition education in this population should be improvement of knowledge.

Nutrition education must start in the early pre-school and kindergarten years, and cannot be delayed until these children will be old enough to understand every aspect of the dietary recommendations. School principals, teachers and food service workers have the opportunity to include nutrition education to the entire school curriculum. For example: efforts should be made to practice selections of low fat and carbohydrate food, yet with acceptable taste; to prepare and taste different fruits and vegetables; to visit food companies and places where food is grown and processed; to teach ways to purchase healthier food in the supermarket; to supply instructions on how to control environments that encourage overeating such as celebrations, restaurants, and to provide special food considerations during vacations.

Nutrition and physical activity information and knowledge should be incorporated into other school classes. When teaching about the holidays, teach about unique foods and meals, when teaching geography, discuss food in different cultures and nations, during mathematics classes, use calculation of food content from food labels, calculation of distance running, or heart rate etc. All these may encourage awareness and knowledge of children to healthy living. Previous nutrition intervention studies in kindergarten children resulted in positive effects on food knowledge and selections [20,21].

Physical activity preferences scores were significantly higher than physical activity knowledge scores and from the nutrition preference scores in the low socio-economic kindergarten children. These results suggest that there is an inherent instinct to prefer physical activity despite the lack of knowledge. The lower physical activity knowledge scores for the low socioeconomic kindergarten children, and the correlation between physical activity knowledge and preferences suggest that in order to promote physical activity, efforts should be made to increase children's knowledge on the importance of increased physical activity and improved fitness on health status. Promotion of physical activity in kindergartens and schools may result not only in reduction of childhood diseases (obesity, diabetes, lipid abnormalities, asthma etc.), but may also increase academic performance, and improved quality of life [22,23].

Nutrition preferences scores were significantly lower among overweight (BMI > 85%) compared to normal weight low socio-economic status kindergarten children. There were no significant differences in nutrition knowledge and/or physical activity knowledge and preferences between the overweight and normal weight children. While a lower nutrition preference score may contribute to the development of overweight and obesity, the results suggest that there might be a gap between knowledge or preference and its actual implementation in overweight kindergarten children. Therefore, efforts should be made to create environments that will encourage obese pre-school and kindergarten children to essentially eat healthier foods and exercise.

Finally, we previously demonstrated gender differences in the moderate-high socio-economic status kindergartners' nutrition and physical activity knowledge and preference scores. Nutrition knowledge and preference scores were significantly higher in females, and physical activity preferences were significantly higher in males. In the present study of children from low socio-economic status, physical activity knowledge and preferences were significantly higher among the males, and while there was a tendency for better nutrition knowledge in the females, it did not reach statistical significance. This appealing finding indicates that the development of gender-specific favorite interests and inclinations occurs as early as the pre-school and kindergarten years, and that socio-economic classes' differences in nutrition and physical activity are already present in these early stages. Therefore, in low-income, kindergarten-aged children, teachers, health educators and health-care providers should make an effort to increase nutrition knowledge in both genders and to improve physical activity knowledge and preferences and encourage exercise especially in girls.

In summary, nutrition and physical activity knowledge scores of low socio-economic kindergarten children were poor and significantly lower compared to moderate-high socio-economic kindergarten children suggesting a possible explanation for the higher prevalence of overweight in this population. Significantly higher scores of physical activity preferences compared to physical activity knowledge may partially compensate for the lack of knowledge in low socio-economic kindergarten children. Nutrition preferences scores were significantly lower among overweight compared to normal weight kindergarten children. Gender-specific differences indicated better physical activity knowledge and preferences in boys. These findings may be useful in developing specific strategies to promote healthier life-style in kindergarten children from different socio-demographic groups, and in designing programs for the prevention and treatment of childhood obesity.

## Abbreviations

PA: physical activity; BMI: body mass index.

## Competing interests

The authors declare that they have no competing interests.

## Authors' contributions

DN and AA conceived the study, participated in its design and coordination, assisted in the statistical analysis and helped to draft the manuscript. DG was responsible for data acquisition and database creation. YM assisted in the data analysis and interpretation and was involved in drafting the manuscript. All authors read and approved the final manuscript.
